# Physical activity disparities across Europe: clustering European regions by health-related physical activity levels

**DOI:** 10.1093/heapro/daab157

**Published:** 2021-10-11

**Authors:** Fernando Lera-Lopez, Rocio Marco

**Affiliations:** 1 Department of Economics, Public University of Navarra, Campus Arrosadía s/n, 31006 Pamplona, Spain; 2 Department of Applied Economy, Universidad Autónoma de Madrid, Ciudad universitaria de Cantoblanco, Francisco Tomás y Valiente Road, number 5, 28049 Madrid, Spain

**Keywords:** physical activity, health, sports participation, European regions, cluster analysis, disparity, taxonomy

## Abstract

In the context of stagnating global levels of physical activity (PA), this study examines the geographical segmentation of PA at the regional level (196 regions) in Europe. Cluster analysis and multinomial logistic regression are applied. Cluster analysis provides a taxonomy of four differentiated groups according to the health-related PA levels of the European regions. This taxonomy shows that there are significant regional disparities among European countries in terms of the regional PA level. The cluster profiles in terms of regional socioeconomic characteristics are described for each group, emphasizing the regional characteristics associated with PA. Regional economic variables, tertiary education and social Internet use are significant variables for characterizing the types of regions. The results emphasize the relevance of a European regional approach for reducing inter-regional PA disparities and improving health through PA in Europe. Practical implications of this research are based on regional European coordination, such as collaborative models of sport infrastructure use, co-financing of inter-regional facilities, mutual physical educational scholar programs and promotion of common inter-regional sport competitions and sporting events. Finally, formal schemes for exchanging of best regional practices to promote health-enhancing PA might increase the perception and the role of PA at the regional level in the European society.

## INTRODUCTION

In spite of the well-established relationship between physical activity (PA) and health (World Health Organization [WHO], 2020), empirical evidence has shown that worldwide inactivity rates have risen during the last decade (Guthold *et al.*, 2018), reaching 46% in Europe (European Commission, 2018). This situation has recently worsened during the COVID-19 pandemic and home confinement in many countries, showing a general decrease in PA levels (Violant-Holz *et al.*, 2020; Trabelsi *et al.*, 2021). Activity data provided by wearable devices, such as Fitbit (Fitbit, 2020) and Garmin (Garmin,2020), have reported, for example, a drastic global decline in total steps during the countryside lockdowns, with higher decreases in countries where home confinement was most extreme. At the same time, a relative increase in exercise interest has been reported in some countries (Ding *et al.*, 2020), arguing that being active is more important post-COVID-19 rather than pre-COVID-19.

These recent trends in PA decline are of great concern because of the impact on increasing healthcare costs through non-communicable diseases, including obesity. Systematic reviews made by Ding *et al.* (Ding *et al.*, 2017) consequently view physical inactivity as a global pandemic with estimated health costs from physical inactivity ranging from 0.3% to 4.6% of national healthcare expenditures, and a total cost to international healthcare systems of $53.8 billion. Recently, Strain *et al.* estimated 3.9 million premature deaths taking place annually due to the lack of PA (Strain *et al.*, 2020).

Traditionally, steps to fight against Europe’s physical inactivity time bomb have been focused on national debates and measures (i.e. Kahlmeier *et al.*, 2015; WHO, 2018; Gelius *et al.*, 2020; Troiano *et al.*, 2020), as well as detailed national analyses of the correlates of PA, and focused on the traditional study of individual characteristics and personal traits (i.e. age, education level, income, etc.; Bauman *et al.*, 2012; Cabane and Lechner, 2015). From a geographical perspective, different studies have included variables at the national level to explain PA and sport. Some papers have considered the positive impact of the provision and condition access of sport infrastructure (Cereijo *et al.*, 2019) and macroeconomic factors like gross domestic product (GDP; i.e. Ruseski and Maresova, 2014). Other articles have investigated the impact of different functional government spending (i.e. health and education expenditures, sport and recreational expenditures) on sports participation (Lera-López *et al.*, 2016) and PA rates in the EU (Szczepaniak, 2020). Some researchers have examined the role of national government quality on European sports participation rates (Wicker and Downward, 2017).

Other studies have tested the disparities among countries in terms of PA levels. In Europe, different works have shown significant differences in the amount of leisure-time PA among the European countries, showing that PA and sport rates decline from north to south and from west to east (Van Tuyckom *et al.*, 2010; Van Tuyckom and Scheerder, 2010; Scheerder *et al.*, 2011). More recently, empirical findings considering the EU-28 have confirmed this geographical divide (Kornbeck, 2013; Gerovasili *et al.*, 2015). These differences have motivated a recent analysis of the PA recommendations applied in the EU countries (Gelius *et al.*, 2020).

Nevertheless, although this problem could be considered as a national issue, some institutions advocate for regional and local approaches to ensure a long-term sustainable impact of PA and emphasize the relevance of regional differences (Mattli *et al.*, 2019) to promote PA in Europe. Only few papers have developed a regional approach in Europe (i.e. Lera-López and Marco, 2018; Clemente Remón *et al.*, 2020) despite regional differences and the role played by regional characteristics in explaining PA (Kokolakakis *et al.*, 2017).

This study tries to overcome this shortcoming by considering a wide set of regions and estimating health-related PA levels in 196 European regions. Following this approach, our main purpose is twofold. On the one hand, we try to classify European regions into differentiated groups according to the health-related PA levels of their citizens. On the other hand, we want to define the main regional socio-economic characteristics that explain this regional classification, identifying the features that are decisive to increase PA among the European regions. To the best of our knowledge, no previous studies have followed this approach of defining the main regional characteristics to explain differences in health-related PA levels.

The rest of this article is organized as follows. Data and the empirical methodology adopted in the article are presented in Data and Methodology. The Results section describes the main results before discussing them and concluding with some policy implications in Discussion and Conclusion.

## DATA AND METHODOLOGY

### Data

The data related to sport and PA employed in our analysis correspond to individual participation from the Special Eurobarometer 472 (European Commission, 2018). The survey covers 28 031 individuals aged 15 years and over in the 28 European Union member states in December 2017. Stratification by individual unit and type of area guarantees the representativeness of the whole territory of the countries surveyed according to the second level of Eurostat *Nomenclature d’Unité Territoriales Statistiques* (NUTs2). This information is regionalized to estimate the regional participation rates at the NUTs2 level, with the exception of data from the UK, Italian and German regions because no information about the NUTs2 level is provided for these countries in the survey. In these countries, data correspond to the NUTs1 level. The total territory under study comprises 196 European regions.

The present study takes the conceptualization of sport and PA suggested by the European Sport Charter (Council of Europe, 2001), given the geographical area of analysis, the European regions. In this charter, “sport means all forms of physical activity which, through casual or organized participation, aim at expressing or improving physical activity fitness and mental well-being, forming social relationships or obtaining results in competition at all levels” (Council of Europe, 2001, n.p.). For PA, we mean PA for recreation or non-sport-related reasons, such as cycling from one place to another, dancing, gardening and walking (European Commission, 2018).

Also, as we want to analyze health-related sport and PA levels, we follow the WHO guidelines for adults aged 18–64 years (WHO, 2010) and applied in a majority of European countries (WHO, 2018; Gelius *et al.*, 2020). Thus, the initial sample is restricted to respondents in this age group, reducing the initial sample of 28 013–19 645 individuals for the calculations of regional sport rates. To aggregate the time dedicated to different intensities, a vigorous activity is worth twice a moderate activity, following the WHO guidelines. Additionally, we have considered a moderate activity as worth twice a walking activity. The individuals are classified in one of four groups according to their answers. People physically active but having an activity level below the WHO guidelines for the working-age population (18–64 years) are classified as *Below-healthy*: below 150 min of moderate-intensity activity or 75 min of vigorous-intensity activity per week or an equivalent combination of both. Individuals who have an activity level meeting the WHO guidelines, but who do not secure additional health benefits, are classified in the *Healthy* group. According to the WHO (WHO, 2010, 2020), extra health benefits can be obtained when adults engage in 300 or more minutes of moderate-intensity activity or 150 min of the vigorous-intensity activity or an equivalent combination of both. Therefore, the third group includes the *Extra-healthy* people, individuals whose activity level meets the extra health benefits requirements. Fourth, individuals who do not practice any physical activity at all are classified as *Non-active*. Supplementary Appendix Table S1 summarizes how individuals are classified according to time and intensity. Similar categorization has been followed by other European studies (i.e. Clemente Remón *et al.*, 2020). Finally, the individual classification has been regionalized, estimating for each region the percentage of non-active individuals as well as individuals with below-healthy, healthy and extra-healthy levels of PA. Table 1 shows an overview of the four PA regional indicators.

**Table 1:** Main descriptive statistics

Variables	Minimum	Maximum	Mean	Median	Standard deviation	Variable description
Non-active	0.000	41.46	9.308	6.799	8.199	Rate of non-active people, in %, only working-age population
Below-healthy	0.000	66.667	25.784	24.597	11.445	Rate of active people below the WHO (2010) guidelines, in %, only working-age population
Healthy	0.000	75.000	19.972	18.662	8.662	Activity rate in % for people meeting the WHO (2010) guidelines, only working-age population
Extra-healthy	0.000	84.000	41.806	43.029	16.375	Activity rate in % for people securing additional health benefits, only working-age population
GDPpc	9300.00	75 900.00	27 861.48	25 550.00	11 080.61	Gross domestic product (GDP) at current market prices purchasing power standard per inhabitant
Unemployment	1.700	29.100	7.908	6.300	5.341	Unemployment rate 15 years and older
Density	3.400	7421.60	340.827	117.050	847.610	Population density (number of inhabitants per square km)
EconActivity	54.200	84.700	73.379	73.550	5.150	Economic activity rate in %, 15–64 years
Agriculture	0.000	46.488	5.680	3.281	6.816	Agriculture, forestry and fishing, employment in %, 15 years and older
Industry	3.863	42.961	17.461	15.957	7.583	Industry, employment in %, 15 years and older
Construction	2.818	11.871	6.799	6.845	1.547	Construction, employment in %, 15 years and older
Services	32.137	93.351	70.192	71.655	11.252	Services, employment in %, 15 years and older
Population <15	11.000	21.300	15.605	15.500	1.966	Population younger than 15 years in %
Population 15–64	59.300	70.200	64.924	65.100	2.456	Population 15–64 years in %
Population >64	11.500	26.300	19.477	19.300	2.928	Population 65 years and over, in %
Tertiary	12.100	57.100	31.396	30.800	9.215	Population aged 25–64 with tertiary education in %
SocialUsers	35.000	85.000	55.714	54.000	10.963	Individuals participating in social networks in %
Poverty&SocialRisk	8.600	48.700	22.301	20.300	8.214	People at risk of poverty or social exclusion, in %

*Note:* All data correspond to 2017.

Other regional-level variables were included in the analysis. Since previous research has documented that participation in sport and PA is affected by economic variables (i.e. Van Tuyckom, 2011; Ruseski and Maresova, 2014; Wicker and Downward, 2017), we have included GDP at current market prices purchasing power standard per inhabitant (GDPpc) and the unemployment rate (Unemployment) at the regional level. In addition, we have included the percentage economic activity rate (EconActivity); the percentage of regional population at risk of poverty or social exclusion (Poverty&SocialRisk); and the regional percentages of employment in agriculture, forestry and fishing (Agriculture), in industry (Industry), in construction (Construction) and in services (Services), following the empirical evidence provided by Kokolakakis *et al.* (Kokolakakis *et al.*, 2017). Since urbanization and agglomeration effects as well as educational levels have previously affected regional and national participation levels (Van Tuyckom, 2011; Kokolakakis *et al.*, 2017; Clemente Remón *et al.*, 2020), we have included population density (Density), the distribution of population by age groups (Population <15, Population 15–64 and Population >64) and population with tertiary education (Tertiary). Finally, we considered the regional percentage of individuals participating in social networks (SocialUsers), since it could be argued that social networks use, and use of the Internet in general, could reduce the amount of time devoted to any PA. Really, it could be claimed that social use of the Internet is also capturing the use of the Internet to some extent. All these demographic and socioeconomic regional variables were taken from Eurostat for the year 2017 (Eurostat, 2020). Table 1 shows the main descriptive statistics.

### Methodology

According to the aim of the research, we employ cluster analysis to explore a taxonomy of the European regions based on their health-related PA levels. The cluster analysis technique aims to maximize the homogeneity of the elements within the cluster while also maximizing the heterogeneity between the clusters. A hierarchical cluster procedure is carried out in a preliminary stage to explore the number of clusters that best fit the data (Johnson and Wichern, 2007; Hair *et al.*, 2014). Once the number of groups is determined, the *K*-means non-hierarchical procedure is applied in a second step to provide a more accurate clustering of the regions (Hair *et al.*, 2014).

The clustering is the qualitative outcome that is subsequently modeled by applying a multinomial logistic regression (MLR). Using a battery of social and economic indicators, the econometric model allows identifying what factors influence the probability of belonging to one of the clusters of regions according to their PA levels. The MLR works as follows. The response variable *Y* takes a discrete set of values reflecting *J* categories. *Y_i_* is the value that indicates the qualitative response for the *i*th individual, the probabilities in a multinomial logit model are (Greene, 2012):

(1) $$\mathrm{Prob}\left(Y_{i}=j\right)=P_{ij}=\frac{\exp({x’}_{i}\beta_{j})}{1+\sum_{g=1}^{J}\exp({x’}_{i}\beta_{g})}$$

where ***x***_*i*_ represents the characteristics of the individual and ***β***_*j*_ is the coefficient vector for the *j*th category of the dependent variable. The log-odds can be computed between any pair of alternatives. Taking *h* as the baseline category, the model consists of *J*−1 logit for the response variable to compare each categorical level to the reference category:

(2) $$ln\left[\frac{P_{ij}}{P_{ih}}\right]={x’}_{i}\beta_{j}\;\mathrm{when}\;j\neq \;h\;$$

Therefore, the odds ratio for alternative *j*, *P_ij_/P_ih_*, will also depend on the *h* alternative used as the baseline category. The maximum-likelihood method was used to estimate the parameters of the model.

## RESULTS

### Clusters of European regions based on health-related physical activity rates

The taxonomy of the regions is developed by carrying out the cluster analysis technique. The input variables are the four indicators that define the health-related PA profile of the European regions: the percentage of non-active, below-healthy, healthy and extra-healthy people in each region. After applying several linkage rules and different distance measures under hierarchical procedures, the results point to a robust four-cluster solution. Next, the clusters’ centroids from the hierarchical solution (using the Ward’s linkage method and the squared Euclidean distance) are taken as initial cluster seeds to run the *K*-means algorithm and get the final clustering solution.

**Fig. 1:**
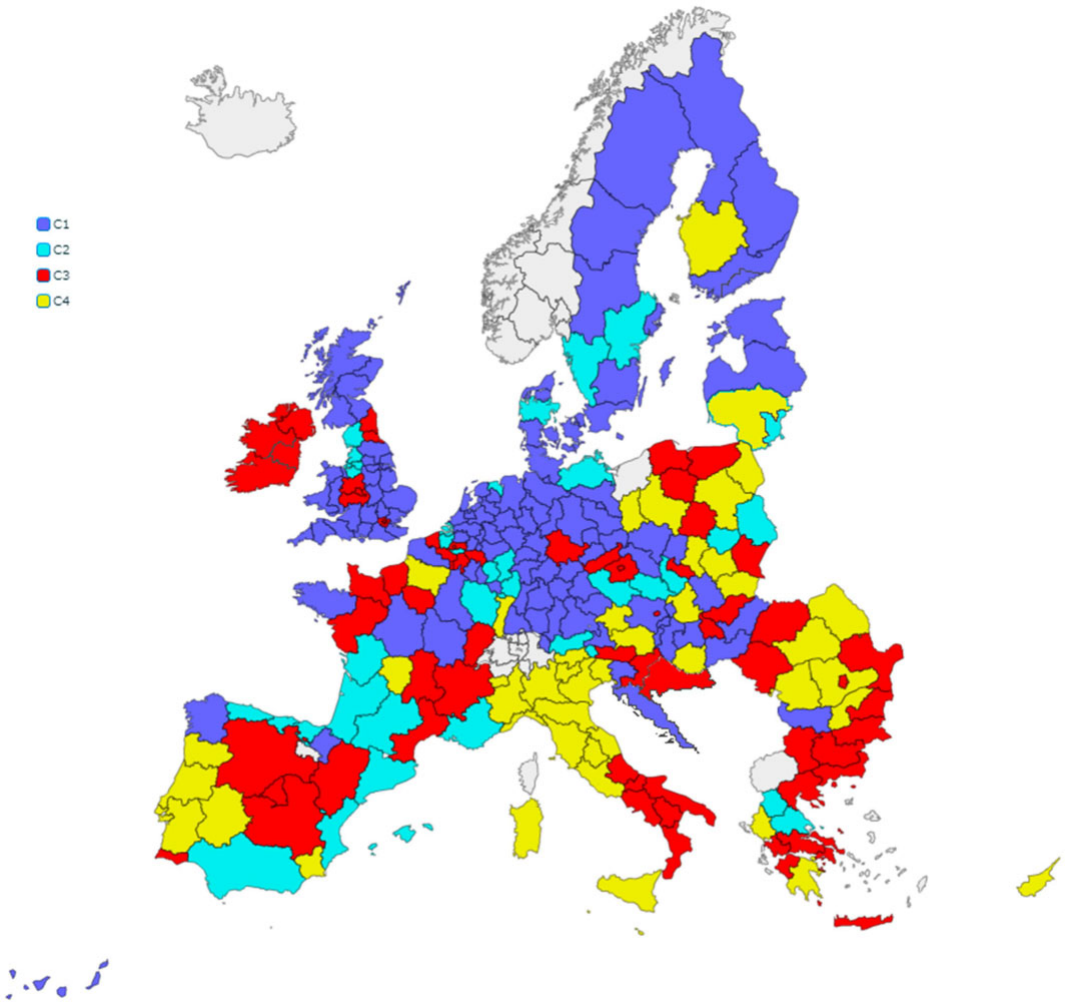
Clustering of the European regions according to their health-related PA levels.

The cluster solution provides a taxonomy of the European regions that can be visualized on the map in Figure 1. C1, the largest group with 73 regions, clusters the regions with a remarkable extra-healthy rate (average rate of 58.0% of the population in this group). C2 is the smallest cluster, 31 regions, and groups the regions outstanding in the healthy rate: on average 32.5% of people meet the healthy guidelines, the largest rate compared to the other clusters. C3 with 57 regions is characterized by having the largest below-healthy rates (an average of 37.3% in this cluster). Consistently, C4 groups the regions with the largest non-active rate (23.3% of the people) and lowest healthy and extra-healthy rates (15.5% and 26.4%, respectively). Supplementary Appendix Table S2 classifies all the European regions according to the four clusters. Regarding the profiles of the groups, C1 is named as the “extra-healthy” cluster, C2 as the “healthy” cluster, C3 as the “below-healthy” cluster, and C4 as the “unhealthy” cluster.

Figure 1 shows that there are significant regional differences among and within European countries in terms of PA levels. Inter-regional disparities within EU countries are important, in particular in countries such as Spain, France, Austria and Poland, which have regions in all clusters. In addition, it is not easy to establish a geographical segmentation of PA levels in the European regions. The traditional north–south and west–east divides shown by previous national studies cannot be completely confirmed at the regional level. Unhealthy regions are mainly concentrated in some southern and eastern areas, but there are some important exceptions.

### Socioeconomic characterization of the clustering on PA rates

Table 2 shows the cluster mean values of some socioeconomic indicators in 2017. For each indicator, the largest value is in bold and the lowest is shaded.

**Table 2:** Socioeconomic characterization of the clustering solution

Variables	C1 extra-healthy	C2 healthy	C3 below-healthy	C4 unhealthy
GDPpc	**31** **532.88**	27 774.19	26 862.28	*21 908.57*
Unemployment	*6.07*	**9.20**	8.94	8.92
Density	336.29	*156.01*	**548.61**	175.59
EconActivity	**76.29**	73.99	70.93	*70.76*
Agriculture	*2.96*	4.73	6.38	**10.97**
Industry	*16.35*	17.09	17.72	**19.68**
Construction	6.77	6.73	*6.67*	**7.12**
Services	**74.23**	71.45	69.22	*62.23*
Population <15	15.68	15.40	**16.09**	*14.84*
Population 15–64	*64.58*	64.59	65.18	**65.51**
Population >64	19.74	**20.03**	*18.73*	19.66
Tertiary	33.29	**34.13**	31.42	*24.99*
SocialUsers	**59.27**	56.35	53.61	*51.14*
Poverty&SocialRisk	*19.40*	20.32	24.52	**26.49**

*Note*: Average values. All indicators correspond to 2017. For each indicator, the largest value is in bold and the lowest is italics.

There is a common pattern by which the less healthy the cluster is (sorted by C1 extra-healthy, C2 healthy, C3 below-healthy and C4 unhealthy), the lower the indicator values. This is true for income (GDPpc), economic activity rate, services and social Internet use, and inversely for agriculture, industry and poverty and social risk indicators. Other indicators do not conform to this pattern: the C3 below-healthy cluster is characterized by a large population density and population below 15 years of age. The C2 healthy cluster holds intermediate positions between the C3 below-healthy and the C1 extra-healthy clusters, but stands out in unemployment, population over 64 years of age and tertiary education.

**Fig. 2:**
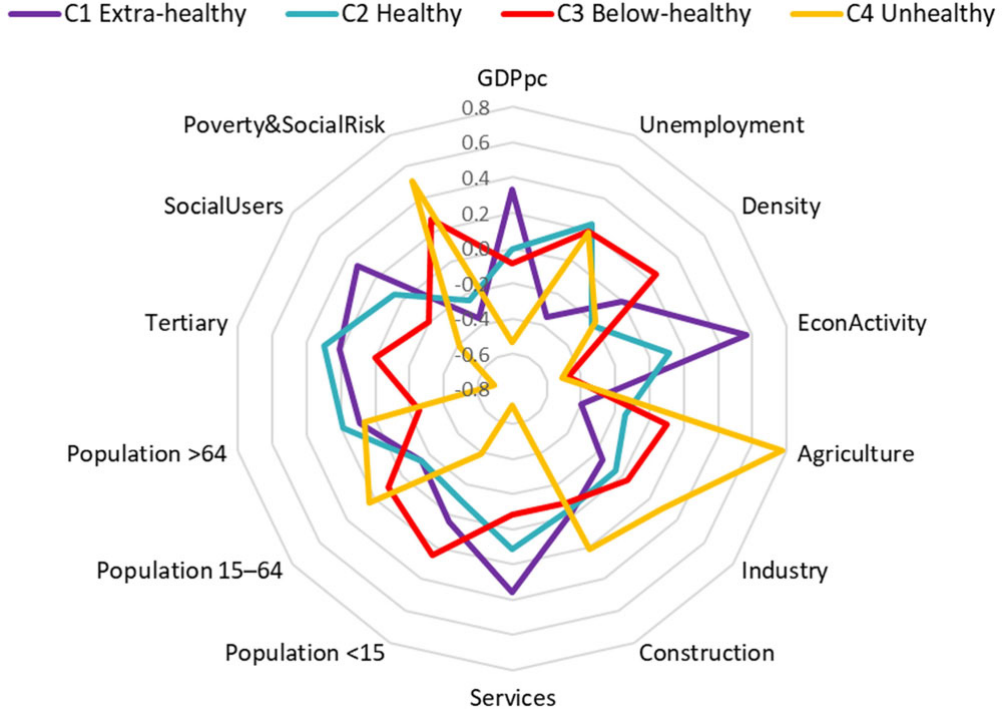
Clustering profile according to (standardized) socioeconomic indicators.

Figure 2 represents the differences among the socioeconomic profiles of the four clusters. The C4 unhealthy cluster is, by far, the most different group compared to the others, standing out in variables such as agriculture and poverty risk. On the other hand, the C1 extra-healthy cluster shows high values for GDPpc, economy activity, services and social Internet use.

One-way analysis of variance (ANOVA) was carried out for each indicator to find out whether differences among clusters are statistically significant. In this bivariate analysis, the categorical factor is the cluster solution that classifies the European regions according to their PA levels in the four groups: C1 extra-healthy, C2 healthy, C3 below-healthy and C4 unhealthy. For each indicator, the null hypothesis stands for no differences across the population group means. Table 3 shows the ANOVA outcomes for those indicators where the univariate normality assumption is not rejected.

**Table 3:** Results of the one-way ANOVA analyses

Variables	*F*	*p*-value	Post hoc multiple comparisons
GDPpc	6.741	0.000	C1 ≠ C4
Unemployment^a^	4.116	0.008	C1 ≠ {C2, C3, C4}
EconActivity	19.589	0.000	{C1, C2} ≠ {C3, C4}
Industry	1.581	0.195	—
Construction	0.663	0.576	—
Services	10.620	0.000	{C1, C2, C3} ≠ C4; C1 ≠ C3
Population <15	3.199	0.025	C3 ≠ C4
Population 15–64	1.544	0.204	—
Population >64	1.865	0.137	—
Tertiary	8.457	0.000	{C1, C2, C3} ≠ C4
SocialUsers^a^	6.084	0.001	C1 ≠ {C3, C4}
Poverty&SocialRisk^a^	8.223	0.000	C1 ≠ C3; {C1, C2} ≠ C4

*Note*: *F* reports the *F*-ratio statistic testing the null hypothesis of equal means. Pairwise multiple comparisons report the clusters pairs with significant mean differences at 5% significance level using the Bonferroni procedure. Tamhane’s T2 procedure is used for those indicators (^a^) where variance homoscedasticity is not accepted.

a *F-*statistic reports the Brown–Forsythe robust test of equality of means for the indicators where the assumption of variance homoscedasticity is not accepted according to the Levene’s statistic test (*α* = 5%).

Regarding the ANOVA outcomes, the regional classification according to PA levels is related to the level of GDPpc, unemployment, economic activity rate, services, population <15, tertiary, social use of the Internet and poverty and social risk at the usual 5% significance level. Pairwise multiple comparisons suggest that the rejection of the null hypothesis is mostly due to the differences between the C4 unhealthy group compared to the others, an outcome that Figure 2 illustrates well.

### Multinomial logistic regression

The model predicts the PA cluster that a region is likely to belong to given the socio-economic indicators used to characterize it. Regarding the regressors, the four economic sector shares add up to 100%, as does the sum of the three population shares, so one indicator of each subset is removed from the explanatory set to avoid perfect multicollinearity in the model. Agriculture is excluded, so this variable acts as the reference for the subset of economics sector weights (industry, construction, services and agriculture). Likewise, population >64 is the reference variable for the population shares (population under 15, population between 15 and 64, and population above 64).

As in a regression model, high collinearity can affect the estimates of the parameters in the MLR. The different criteria used to address multicollinearity (tolerance and variance inflation factor) discard this problem in the model specification.

Regarding the overall model fitting, the LR (likelihood ratio) test ($\chi_{36}^{2}$ = 114.64, *p* < 0.001), as well as the Cox and Snell’s (0.44) and Nagelkerke’s (0.48) pseudo-*R^2^* values point to the substantive significance of the model. Regarding the classification accuracy, the percentage of regions correctly predicted by the MLR is 54.6%. The model demonstrates an acceptable level of practical significance, as the hit ratio exceeds in more than one-fourth of cases the maximum chance criterion [(73/196) × 1.25 = 46.5%] (Hair *et al.*, 2014). We also assessed the independence of irrelevant alternatives assumption by carrying out the Hausman–McFadden specification test. The tests are not conclusive, as we get different results depending on the category considered (Supplementary Appendix Table S4). Table 4 shows the parameter estimates for the MLR final model. The pair comparisons take the C4 unhealthy cluster as the baseline category.

**Table 4:** Multinomial logistic regression: parameter estimates

				95% CI for *Exp(b)*	
Variables	*b*	Standard Error	*Exp(b)*	Lower bound	Upper bound
C3 below-healthy versus C4 unhealthy					
GDPpc	0.000	(0.000)	1.000	1.000	1.000
Unemployment	−0.028	(0.052)	0.973	0.878	1.078
Density	0.000	(0.000)	1.000	0.999	1.001
EconActivity	−0.049	(0.059)	0.952	0.848	1.069
Industryª	0.076	(0.047)	1.080	0.984	1.184
Constructionª	−0.144	(0.157)	0.866	0.637	1.178
Servicesª	0.050	(0.037)	1.052	0.978	1.131
Population <15^b^	0.341	(0.139)**	1.406	1.072	1.845
Population 15–64^b^	−0.033	(0.104)	0.967	0.788	1.187
Tertiary	0.087	(0.041)**	1.091	1.006	1.182
SocialUsers	0.002	(0.028)	1.002	0.948	1.059
Poverty&SocialRisk	0.032	(0.034)	1.032	0.965	1.104
Intercept	−7.844	(10.253)			
C2 healthy versus C4-unhealthy					
GDPpc	0.000	(0.000)	1.000	1.000	1.000
Unemployment	0.100	(0.064)	1.105	0.975	1.253
Density	−0.002	(0.001)*	0.998	0.995	1.000
EconActivity	0.132	(0.079)*	1.141	0.979	1.331
Industry^a^	0.097	(0.076)	1.102	0.949	1.280
Construction^a^	−0.283	(0.190)	0.754	0.519	1.094
Services^a^	0.106	(0.064)	1.112	0.980	1.261
Population <15^b^	0.057	(0.158)	1.059	0.777	1.443
Population 15–64^b^	0.019	(0.122)	1.019	0.802	1.295
Tertiary	0.099	(0.046)**	1.105	1.008	1.210
SocialUsers	0.075	(0.035)**	1.078	1.008	1.154
Poverty&SocialRisk	−0.120	(0.057)**	0.887	0.793	0.992
Intercept	−26.618	(12.486)**			
C1 extra-healthy versus C4 unhealthy					
GDPpc	0.000	(0.000)	1.000	1.000	1.000
Unemployment	−0.037	(0.063)	0.964	0.852	1.090
Density	−0.001	(0.000)	0.999	0.998	1.000
EconActivity	0.176	(0.067)***	1.192	1.045	1.361
Industry^a^	0.125	(0.070)*	1.133	0.989	1.299
Construction^a^	−0.222	(0.173)	0.801	0.571	1.124
Services^a^	0.145	(0.061)**	1.156	1.025	1.303
Population <15^b^	0.134	(0.143)	1.144	0.863	1.515
Population 15–64^b^	−0.049	(0.110)	0.953	0.768	1.182
Tertiary	0.030	(0.041)	1.030	0.951	1.116
SocialUsers	0.054	(0.030)*	1.055	0.995	1.119
Poverty&SocialRisk	−0.029	(0.043)	0.972	0.892	1.058
Intercept	−26.825	(11.359)**			

*Note*: Model $\chi_{36}^{2}$ = 114.637, *p* < 0.001; pseudo *R^2^* = 0.443 (Cox and Snell), 0.476 (Nagelkerke).

a Agriculture is the reference variable for the economic sectors set (Industry, Construction, Service and Agriculture).

b Population >64 is the reference variable for the population share set (population <15, population 15–64 and population >64).

***, ** and * denote significance at 1% (*p* < 0.01), 5% (*p* *<* 0.05) and 10% (*p* < 0.10), respectively.

Population under age 15 and tertiary education help to predict whether a region belongs to the C3 below-healthy or C4 unhealthy cluster. Both odds ratios, *Exp(b)*, are >1 (values of 1.406 and 1.091, respectively), meaning that as the indicator increases, the odds of a region being C3 increase. That is, the larger the proportion of people possessing tertiary education and being under 15 in age, the more likely a region is to be a below-healthy rather than an unhealthy region.

An increase in tertiary education (odds ratio 1.105), social use of the Internet (1.078) and economic activity (1.141) helps to generate a switch from the C4 unhealthy to the C2 healthy cluster, while a rise in population density or people under poverty and social risk decreases the odds of being a C2 healthy region in favor of a C4 unhealthy region (odds ratios 0.998 and 0.887, respectively).

Finally, what significantly predicts whether a region is an extra-healthy (C1) or an unhealthy region (C4) is the economic activity rate, the economic structure and social Internet use. Specifically, a 1% point rise in the economic activity rate increases by 1.192 the odds of being C1 rather than C4. Larger industry or services sectors, to the detriment of agriculture, increase the odds of being extra-healthy by 1.113 and 1.156, respectively. Use of the Internet also makes it more likely to be an extra-healthy region (odds ratio 1.055).

To sum up, younger population, larger proportion of having a tertiary education and fewer people at risk of poverty and social exclusion are the main factors helping to give “small and medium jumps” (from being an unhealthy region to becoming a below-healthy or a healthy region). Meanwhile, increases in social use of the Internet, economic activity rate and reduced agriculture size (in favor of services and industry sectors) are the key factors that support the “big jump” from the unhealthy cluster to the extra-healthy. GDP per capita, unemployment rate, size of the construction sector and population aged 15–64 are not significant in explaining the probability of belonging to any of the PA clusters in the MLR model.

## DISCUSSION AND CONCLUSION

This study analyses the geographical distribution of health-related PA levels in 196 regions in the European Union. The use of sport data aggregated by NUTs2 level allows for the formulation of policy recommendations at the EU regional level, which could provide valuable insights for promoting PA. Applying the cluster technique, a taxonomy of four different European regions is obtained. A set of 35 regions (Cluster C4) could be classified as unhealthy regions, including high percentages of the populations of Bulgaria, Cyprus, Greece, Italy, Malta, Poland, Portugal, and Romania, for example. In contrast, a set of 73 regions (Cluster C1) are categorized by extra healthy regions, belonging mainly to the Baltic countries, Denmark, Germany, the Netherlands and Sweden, among others. Supplementary Appendix Table S3 classifies for every country the regions in each of the clusters.

Some interesting implications could be highlighted considering the clusters obtained. First, there are significant regional disparities among European countries in terms of the regional PA levels, confirming previous studies made in England (Kokolakakis *et al.*, 2017) and across the whole of Europe (Lera-López and Marco, 2018). In addition, the results show that it is not easy to establish a geographical stratification of PA levels in the European regions. The traditional north–south and west–east divides shown by previous national studies (Van Tuyckom *et al.*, 2010; Van Tuyckom and Scheerder, 2010; Scheerder *et al.*, 2011) cannot be completely confirmed at the regional level. Unhealthy regions are mainly concentrated in some southern and eastern areas, but there are some important exceptions. A significant increase in PA levels in these less healthy regions of Europe, to achieve at least the level of PA recommended by the WHO, could have a significant impact on health and subjective well-being of individuals. In this sense, the regional approach allowed us to provide a more nuanced picture of the national segmentation shown in the EU by the previous country-level empirical studies.

Second, we have explored the main regional socio-economic characteristics that explain the differences among the groups of regions. Regional economic variables such as GDPpc, economy activity, economic structure of sectors, tertiary education and social Internet use are significant variables for classifying European regions according to the health-related PA levels, showing that the profile of the unhealthy regions (Cluster C4) statistically differs from the rest of the clusters. Previous studies have emphasized the relevance of regional economic variables (Kokolakakis *et al.*, 2017; Wicker and Downward, 2017), but no previous evidence has been obtained about the role played by other economic variables such as the relevance of the different sectors of the economy and poverty. The influence of regional social Internet use on PA levels deserves special attention. Its influence could be related to the fact that part of the social use of the Internet is closely associated with sports news, and through social networks, people could be inspired to become involved in sport and PA.

**Fig. 3:**
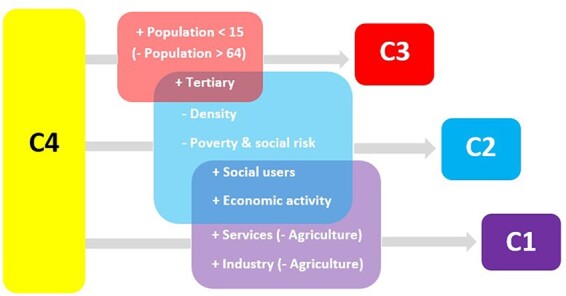
Summary of determinant factors for improving PA levels.

Third, we have established different strategies, based on the determining characteristics of the regions, to move regions from the unhealthy cluster (C4) to the healthier categories. Figure 3 summarizes these strategies. A first strategy is based on becoming active. In this case, any policy to increase the population with tertiary education would increase the level of PA among the citizens of the regions. Nevertheless, if the purpose is to meet the WHO guidelines on PA levels, boosting social Internet use and economic activity is also very relevant. Finally, if the region wants to improve health-related PA levels above the WHO standards, an increase in the economic activity rate, the social use of the Internet and an increase of the services and industry sectors should be considered. These results emphasize the relevance of education, economic variables and the Internet, but the combination of variables differs depending on the PA level that is targeted.

Some interesting practical implications for sport and health policymakers can be described. Mainly, the results emphasize the necessity to develop a regional approach across the EU based on regional coordination, collaborative models of action and co-financing, including specific policies, according to the taxonomy of regions shown in this article, for promoting PA in the less healthy regions. In this context, the EU could play a leadership role in harmonizing the level of PA through regional initiatives, following the framework of European cooperation in the field of sport (Council of the European Union, 2020), and turning PA and sport into a real instrument for EU cohesion policy. For example, by means of EU funding programs, such as the European Structural and Investment Funds, the building of inter-regional sport infrastructure could be promoted, to be used by citizens of border regions belonging to different countries, in the context of the positive impact of regional sporting facilities on PA levels (Kokolakakis *et al.*, 2017; Cereijo *et al.*, 2019). Besides, considering the previous existence of positive PA spillovers at the regional level in the EU (Lera-López and Marco, 2018), cross-regional sport cooperation based on an inter-regional use of sporting facilities could reduce regional disparities in PA. In addition, other initiatives for boosting cross-regional sport cooperation could be considered, such as mutual physical education programs in schools, common amateur sport competitions and inter-regional sporting events, particularly among young people within national border regions. In addition, the Erasmus+ program could be used as a way to promote the role of sport and PA in society, particularly at the regional level, through practical initiatives with regional sport stakeholders to increase regional sport cooperation within the Member States. Finally, some measures such as the SHARE initiative (European Commission, 2021) should be encouraged as a scheme for the exchange of best regional practices to promote health-enhancing PA.

To sum up, these results emphasize the relevance of a European regional approach for a better understanding of the disparities in health-related PA levels in Europe. These differences might be a result of the progressive process of European decentralization in policy decisions about health care, including PA. As Vos *et al.* (Vos *et al.*, 2016) have described, an increasing process of decentralization of sport policies has been widely developed in many European countries. However, it could be argued that if PA policies are implemented at the regional and local levels, differences in aims and funding would be a factor producing divergence in PA rates among regions within countries. In the context of the EU, this situation could be of concern, considering the positive externalities produced by higher sport and PA rates such as the improvement of health, the creation of employment and the enhancement of social cohesion (Vos *et al.*, 2016). In addition, previous empirical evidence has shown the existence of positive spatial spillovers for sports and PA rates at the regional level in the EU (Lera-López and Marco, 2018), suggesting that this practice seems to transcend national boundaries and requires a more general perspective than the national approach.

This study has certain limitations that could be addressed in further research. Like other empirical studies based on official surveys and databases, this research is limited to the available data. Unfortunately, some regional variables of interest such as government spending on sport and PA policies and environmental factors are not available from Eurostat. More detailed information about the four domains of PA (leisure time, work, active transportation and household) might be very interesting to adequately tackle the European regional PA disparities described above. Further research should also develop a longitudinal analysis to determine the evolution over time of these inequalities in PA levels among the European regions. Finally, it could be very useful to examine the effects of the COVID-19 pandemic on the regional PA levels in the EU.

## SUPPLEMENTARY MATERIAL

Supplementary material is available at *Health Promotion International* online.

## FUNDING 

This work was supported by the Spanish Ministerio de Economia, Industria y Competitividad and FEDER [grant number ECO2017-86305-C4-4-R AEI/FEDER to F.L.].

## CONFLICT OF INTEREST

The authors declare that there is no conflict of interest.

## Supplementary Material

daab157_Suplementary_Material
