# Supplementary material for: Physical activity disparities across Europe: clustering European regions by health-related physical activity levels
Source: Health Promot Int. 2021 Oct 11;37(2):daab157. doi: 10.1093/heapro/daab157 (PMC9053455; doi:10.1093/heapro/daab157)
Supplement: daab157_Suplementary_Material [file daab157_suplementary_material.docx]

**Appendix**

| *Table A1. Individual classification according to the WHO guidelines* | | |
| --- | --- | --- |
|  | Intensity | |
|  | Moderate | Vigorous |
| Non-active | *t* = 0 | *t* = 0 |
| Below-healthy | 0 < *t* < 150 | 0 < *t* < 75 |
| Healthy | 150 ≤ *t* < 300 | 75 ≤ *t* < 150 |
| Extra-healthy | *t* ≥ 300 | *t* ≥ 150 |
| Note: *t* = minutes per week. | | |

*Table A2. Regional classification according to PA rates*

| **C1 Extra-healthy group** | |  | |  | |
| --- | --- | --- | --- | --- | --- |
| AT11 Burgenland | DED Sachsen | | HR03 Jadranska Hrvatska | | SE11 Stockholm |
| AT12 Niederoesterreich | DEE Sachsen-Anhalt | | HU21 Central/North Transdanubia | | SE21 Smaland med Oearna |
| AT32 Salzburg | DEF Schleswig-Holstein | | HU22 West Transdanubia | | SE22 Sydsverige |
| AT34 Vorarlberg | DK01 Hovedstaden | | HU32 North Great Plain | | SE31 Norra Mellansverige |
| BE21 Antwerpen | DK02 Sjaelland | | HU33 South Great Plain | | SE32 Mellersta Norrland |
| BE22 Limburg | DK03 Syddanmark | | LU LUXEMBOURG | | SE33 Oevre Norrland |
| BE34 Luxembourg | DK05 Nordjylland | | LV LATVIA | | SI04 Zahodna Slovenija |
| BG31 Severozapaden | EE ESTONIA | | NL12 Friesland | | SK01 Bratislavsky kraj |
| CZ05 Severovychod | ES11 Galicia | | NL13 Drenthe | | SK03 Stredne Slovensko |
| DE1 Baden-Wuerttemberg | ES22 Navarra | | NL21 Overijssel | | UKE Yorkshire and The humber |
| DE2 Bayern | ES70 Canarias | | NL22 Gelderland | | UKF East midlands |
| DE3 Berlin | FI1B Helsinki-Uusimaa | | NL23 Flevoland | | UKH East of England |
| DE4 Brandenburg | FI1C Etela-Suomi | | NL31 Utrecht | | UKJ South East |
| DE5 Bremen | FI1D Pohjois- ja Ita-Suomi | | NL32 Noord-Holland | | UKK South West |
| DE6 Hamburg | FRF2 Champagne-Ardenne | | NL33 Zuid-Holland | | UKL Wales |
| DE7 Hessen | FRB0 Centre | | NL41 Noord-Brabant | | UKM Scotland |
| DE9 Niedersachsen | FRC1 Bourgogne | | NL42 Limburg | |  |
| DEA Nordrhein-Westfalen | FRE1 Nord - Pas de Calais | | PL51 Dolnoslaskie | |  |
| DEC Saarland | FRH0 Bretagne | | PL52 Opolskie | |  |

| **C2 Healthy group** | |  | |  | |
| --- | --- | --- | --- | --- | --- |
| AT33 Tirol | DK04 Midtjylland | | ES53 Baleares | | NL11 Groningen |
| BE23 Oost-Vlaanderen | EL13 Ditiki Makedonia | | ES61 Andalucia | | NL34 Zeeland |
| BE31 Brabant Wallon | EL14 Thessalia | | FRF3 Lorraine | | PL72 Swietokrzyskie |
| CZ03 Jihozapad | ES12 Asturias | | FRI3 Poitou-Charentes | | PL43 Lubuskie |
| CZ06 Jihovychod | ES13 Cantabria | | FRI1 Aquitaine | | SE12 Oestra Mellansverige |
| CZ07 Stredni Morava | ES21 Pais Vasco | | FRJ2 Midi-Pyrenees | | SE23 Vaestsverige |
| DE8 Mecklenburg-Vorpommern | ES51 Cataluna | | FRL0 Provence-Alpes-Cote d-Azur | | UKD North West |
| DEB Rheinland-Pfalz | ES52 Valencia | | LT01 Sostines regionas | |  |

| **C3 Below-healthy group** | |  | |  | |
| --- | --- | --- | --- | --- | --- |
| AT13 Wien | CZ08 Moravskoslezsko | | FRC2 Franche-Comte | | PL62 Warminsko-mazurskie |
| AT21 Kaernten | DEG Thueringen | | FRG0 Pays de la Loire | | PL63 Pomorskie |
| BE10 Brussels hoofdstedelijk gewest | EL11 Anatoliki Makedonia, Thraki | | FRK2 Rhone Alpes | | PT15 Algarve |
| BE24 Vlaams Brabant | EL12 Kentriki Makedonia | | FRK1 Auvergne | | RO11 North-West |
| BE25 West-Vlaanderen | EL23 Ditiki Ellada | | FRJ1 Languedoc-Roussillon | | RO22 South-East |
| BE32 Hainaut | EL24 Sterea Ellada Evia | | HR04 Kontinentalna Hrvatska | | RO32 Bucharest |
| BE33 Liege | EL30 Attiki | | HU11 & HU12 Budapest + Pest | | RO42 West |
| BE35 Namur | EL43 Kriti | | HU31 North Hungary | | SI03 Vzhodna Slovenija |
| BG33 Severoiztochen | ES24 Aragon | | IE04 Northern and Western | | UKC North East |
| BG34 Yugoiztochen | ES30 Madrid | | IE06 Eastern and Midland | | UKG West Midlands |
| BG41 Yugozapaden | ES41 Castilla/Leon | | IE05 Southern | | UKI London |
| BG42 Yuzhen tsentralen | ES42 Castilla/La Mancha | | ITF Sud | | UKN Northern Ireland |
| CZ01 Praha | FR10 Ile de France | | PL71 Lodzkie | |  |
| CZ02 Stredni Cechy | FRD2 Haute Normandie | | PL82 Podkarpackie | |  |
| CZ04 Severozapad | FRD1 Basse Normandie | | PL61 Kujawsko-pomorskie | |  |

| **C4 Unhealthy group** | |  | |  | |
| --- | --- | --- | --- | --- | --- |
| AT22 Steiermark | FRE2 Picardie | | MT MALTA | | PT17 Lisboa |
| AT31 Oberoesterreich | FRF1 Alsace | | PL92 Mazowieckie | | PT18 Alentejo |
| BG32 Severen tsentralen | FRI2 Limousin | | PL21 Malopolskie | | RO12 Central |
| CY REPUBLIC OF CYPRUS | HU23 South Transdanubia | | PL22 Slaskie | | RO21 North-East |
| EL21 Ipiros | ITC Nord-Ovest | | PL81 Lubelskie | | RO31 South |
| EL25 Peloponnissos | ITG Isole | | PL84 Podlaskie | | RO41 South-West |
| ES43 Extremadura | ITH Nord-Est | | PL41 Wielkopolskie | | SK02 Zapadne Slovensko |
| ES62 Murcia | ITI Centro | | PT11 Norte | | SK04 Vychodne Slovensko |
| FI19 Lansi-Suomi | LT02 Vidurio ir vakaru Lietuvos regionas | | PT16 Centro | |  |

| *Table A3. Cluster membership by country: number and % of regions in each cluster* | | | | | | | | | | |
| --- | --- | --- | --- | --- | --- | --- | --- | --- | --- | --- |
| Country | C1 extra-healthy | | | C2 healthy | | C3 below-healthy | | C4 unhealthy | | Num. of regions |
|  | Num. | | % | Num. | % | Num. | % | Num. | % |  |
| Austria | | 4 | 44.4% | 1 | 11.1% | 2 | 22.2% | 2 | 22.2% | 9 |
| Belgium | | 3 | 27.3% | 2 | 18.2% | 6 | 54.5% | 0 | 0.0% | 11 |
| Bulgaria | | 1 | 16.7% | 0 | 0.0% | 4 | 66.7% | 1 | 16.7% | 6 |
| Czech Rep. | | 1 | 12.5% | 3 | 37.5% | 4 | 50.0% | 0 | 0.0% | 8 |
| Germany | | 13 | 81.3% | 2 | 12.5% | 1 | 6.3% | 0 | 0.0% | 16 |
| Denmark | | 4 | 80.0% | 1 | 20.0% | 0 | 0.0% | 0 | 0.0% | 5 |
| Greece | | 0 | 0.0% | 2 | 20.0% | 6 | 60.0% | 2 | 20.0% | 10 |
| Spain | | 3 | 18.8% | 7 | 43.8% | 4 | 25.0% | 2 | 12.5% | 16 |
| Finland | | 3 | 75.0% | 0 | 0.0% | 0 | 0.0% | 1 | 25.0% | 4 |
| France | | 5 | 23.8% | 5 | 23.8% | 8 | 38.1% | 3 | 14.3% | 21 |
| Croatia | | 1 | 50.0% | 0 | 0.0% | 1 | 50.0% | 0 | 0.0% | 2 |
| Hungary | | 4 | 57.1% | 0 | 0.0% | 2 | 28.6% | 1 | 14.3% | 7 |
| Ireland | | 0 | 0.0% | 0 | 0.0% | 3 | 100% | 0 | 0.0% | 3 |
| Italy | | 0 | 0.0% | 0 | 0.0% | 1 | 20.0% | 4 | 80.0% | 5 |
| Lithuania | | 0 | 0.0% | 1 | 50.0% | 0 | 0.0% | 1 | 50.0% | 2 |
| Netherlands | | 10 | 83.3% | 2 | 16.7% | 0 | 0.0% | 0 | 0.0% | 12 |
| Poland | | 2 | 13.3% | 2 | 13.3% | 5 | 33.3% | 6 | 40.0% | 15 |
| Portugal | | 0 | 0.0% | 0 | 0.0% | 1 | 20.0% | 4 | 80.0% | 5 |
| Romania | | 0 | 0.0% | 0 | 0.0% | 4 | 50.0% | 4 | 50.0% | 8 |
| Sweden | | 6 | 75.0% | 2 | 25.0% | 0 | 0.0% | 0 | 0.0% | 8 |
| Slovenia | | 1 | 50.0% | 0 | 0.0% | 1 | 50.0% | 0 | 0.0% | 2 |
| Slovakia | | 2 | 50.0% | 0 | 0.0% | 0 | 0.0% | 2 | 50.0% | 4 |
| United Kingdom | | 7 | 58.3% | 1 | 8.3% | 4 | 33.3% | 0 | 0.0% | 12 |
| *Single-region countries* | | | | | | | | | | |
| Cyprus | | 0 | 0.0% | 0 | 0.0% | 0 | 0.0% | 1 | 100% | 1 |
| Estonia | | 1 | 100% | 0 | 0.0% | 0 | 0.0% | 0 | 0.0% | 1 |
| Luxembourg | | 1 | 100% | 0 | 0.0% | 0 | 0.0% | 0 | 0.0% | 1 |
| Latvia | | 1 | 100% | 0 | 0.0% | 0 | 0.0% | 0 | 0.0% | 1 |
| Malta | | 0 | 0.0% | 0 | 0.0% | 0 | 0.0% | 1 | 100% | 1 |
| *Totals* | 73 | | 37.2% | 31 | 15.8% | 57 | 29.1% | 35 | 17.9% | 196 |
| *Note*: NUTs2 for all countries except Italy, UK and DE (NUTs1) | | | | | | | | | | |

| *Table A4. Hausman-McFadden Independence of Irrelevant Alternatives tests* | | | | |
| --- | --- | --- | --- | --- |
| Omitted | *χ^2^* | df | *P* > *χ^2^* | evidence |
| C1 | -51.83 | 11 | 1.000 | for H_o_ |
| C2 | 67.28 | 12 | 0.000 | against H_o_ |
| C3 | -34.26 | 11 | 1.000 | for H_o_ |
| C4 | -52.1 | 21 | 1.000 | for H_o_ |
| *Note:* *χ*^2^ < 0 denotes that the estimated model does not meet asymptotic assumptions of the test | | | | |
